# Research on the facile regeneration of degraded cathode materials from spent LiNi_0.5_Co_0.2_Mn_0.3_O_2_ lithium-ion batteries

**DOI:** 10.3389/fchem.2024.1400758

**Published:** 2024-04-30

**Authors:** Chen Yang, Yujia Hao, Jiayi Wang, Mingdao Zhang, Li Song, Jiaan Qu

**Affiliations:** School of Environmental Science and Engineering, Jiangsu Key Laboratory of Atmospheric Environment Monitoring and Pollution Control, Jiangsu Collaborative Innovation Center of Atmospheric Environment, Nanjing University of Information Science and Technology, Nanjing, Jiangsu, China

**Keywords:** spent lithium-ion batteries, cathode material, recycle, regeneration, electrochemical performance, ternary lithium-ion batteries

## Abstract

Rational reusing the waste materials in spent batteries play a key role in the sustainable development for the future lithium-ion batteries. In this work, we propose an effective and facile solid-state-calcination strategy for the recycling and regeneration of the cathode materials in spent LiNi_0.5_Co_0.2_Mn_0.3_O_2_ (NCM523) ternary lithium-ion batteries. By systemic physicochemical characterizations, the stoichiometry, phase purity and elemental composition of the regenerated material were deeply investigated. The electrochemical tests confirm that the material characteristics and performances got recovered after the regeneration process. The optimal material was proved to exhibit the excellent capacity with a discharge capacity of 147.9 mAh g^−1^ at 1 C and an outstanding capacity retention of 86% after 500 cycles at 1 C, which were comparable to those of commercial NCM materials.

## 1 Introduction

Since the commercialization in 1990s, lithium-ion batteries (LIB) are popular in the fields of consumer electronics, energy storage systems and electric vehicles (EVs), due to the high energy density, long cycle life and flexible dimensions. Driven by the fast development of EVs in this decade, the LIB market demand has been rapidly expanding, and is expected to drastically rise to 87.5 billion dollars in 2027 (Li et al., 2021). In the LIBs market, the most widely used cathode materials are LiFePO_4_ (LFP) and ternary material LiNi_*x*_Co_*y*_Mn_z_O_2_, (NCM). With the rapid popularization of EVs in the global world, large numbers of LIBs have come into service. However, the power LIBs have only a limited service life of 8–10 years for the EVs (Dinger et al., 2010). It is worth mentioning that the NCM batteries dominate the powder battery market of EVs before 2021. Therefore, in the next 10 years, large amounts of NCM-type LIBs will get retired from the EVs. On the one hand, the abandonment of these LIBs will seriously pollute the ecological environment. Especially, the cathode materials in NCM batteries contain lots of metals such as Ni, Co, Mn, which are harmful to water, atmosphere and soil. On the other hand, the LIB industry is facing a crisis of resource shortage, because the overuse of the valuable Li, Ni Co, and Mn in the past decade, which are needed to be recycled (Roy et al., 2022). Therefore, clean and efficient recovery technologies are needed for sustainable development of LIBs.

Lots of efforts have been devoted to recycling the spent NCM LIBs. The hydrometallurgical process is currently the most general strategy, including a pre-treatment process, leaching process and the recovery process of the constituent metals (Kim et al., 2021). The pre-treatment process mainly aims at detaching the active materials from the collector, usually including discharging, dismantling, separating, *etc.* Yao et al. (2018). After the pre-treatment process, a leaching approach was carried out to transform the constituent elements into the metallic ions. The last step is getting the relevant metallic compounds from the leaching solutions, usually through the ways of evaporation, designed precipitation reactions, electrodeposition, new-type chemical reaction, *etc.* Gao et al. (2018), Gupta et al. (2022), Grima-Carmena et al. (2023), Gu et al. (2023), Ilyas et al. (2023), Qu et al. (2024).

The above recovery method is actually to enrich and separate the metal elements from the leaching solution through precipitation or extraction, and then to recycle them in the form of metal compounds (Jin et al., 2022). However, due to the similar chemical properties of Ni, Co, Mn ions, it is difficult to separate them, which need more procedures to get the pure products. Afterwards, the resultant metallic compounds are further used as the precursors for the synthesis of new ternary lithium materials. Obviously, these traditional ways are time-consuming and energy-intensive.

Therefore, directly regenerating valuable metals from the leaching solution into ternary lithium cathode materials is more attractive, which is thought to be an economic and efficient recycling method, maximizing the recovery value. In these years, some new strategies have been proposed by the scientists to simplify the recycling process of spent LIBs and to improve the economic benefit. Bai et, al. reported a novel leaching and precipitating system in waste NCM materials of LIBs, efficiently obtaining the Ni, Co, Mn and Li intermediates, which can be used to regenerate the NCM cathode material (Bai et al., 2023). He et al made use of the deep eutectic solvents (DESs) to leach the metal components from spent NCM LIBs. The resultant leaching solution was subjected to a co-precipitation reaction and high-temperature calcination, getting the regenerated LiNi_0.5_Co_0.2_Mn_0.3_O_2_ material (He et al., 2023). These reported works inspired us to directly regenerate the NCM material from the spent LIBs. However, there are several problems needed to be solved, such as high lost, complicated steps and deficient purity (Xiao et al., 2019; Zhou et al., 2024). Extracting the Ni, Co, Mn and Li from the spent NCM materials and efficiently transforming them into regenerated NCM materials by the designed stoichiometric ratio is not easy to realize. Another problem is the insufficient performance of the generated NCM materials compared with that of commercial new counterpart (He et al., 2023), thus hindering the wide applications.

In this work, a facile synthesis approach was developed to regenerate the ternary LiNi_0.5_Co_0.2_Mn_0.3_O_2_ (NCM523) material from the leaching solution of spent cathode materials. By treating the waste NCM523 powders with organic acid, we obtain the leaching solution of metal ions. The solid precursor was obtained by subjecting the leaching solution to a precipitation process. The precipitates were directly used as the precursors of synthesizing the regenerated NCM523 material with a solid-state calcination method. The regenerated products were proved to possess the same constitutes, properties and nice electrochemical performances compared to the commercial NCM materials.

## 2 Experiment

### 2.1 Pretreatment

The waste NCM battery were put into a saturated salt solution to get completely discharged and then manually disassembled in an argon-filled glove box. The NCM powders could be peeled from the cathode plate by ultrasonic treatment in water. The powders were further calcined in the muffle furnace at 650°C to remove the PVDF and conductive carbon. Afterwards, the NCM powders were leached with a mixture of formic acid (2 vol%) and citric acid (0.5 mol/L). After filtration, the leaching solution was obtained.

### 2.2 Regeneration

In order to get the metallic precursor, 0.55 mol/L oxalic acid solution was used as the precipitant adding into the leaching solution, which was further aged at 50°C. The precipitates were possessed after a filtration process and then dried at 80°C overnight to obtain the ternary metal oxalate compounds. The as-prepared metal oxalate compounds were mixed with a certain proportion of lithium carbonate by ball milling and were used as the precursor of the regenerated NCM materials. At last, the mixture was calcined in the furnace, with the first heating temperature of 500°C for 5 h, and the second heating temperature for 12 h at a ramping rate of 5°C min^-1^. After natural cooling, we can get the regenerated NCM523 cathode material, noted as R-NCM-X, where X represents the heating temperature. If not noted, R-NCM refers to R-NCM-850.

### 2.3 Battery assembly and electrochemical measurements

The cathode electrodes were made up of active material powder, PVDF and conductive carbon (Ketjen Black), coated on aluminum foil with the mass ratio of 8:1:1. The electrochemical performances were tested in the CR2032 coin-type half cells using the Li disc as the anode, 1 M LiPF_6_ in EC/DEC (1:1 vol%) as the electrolyte and a Celegard 2400 membrane as the separator. Cyclic voltammetry (CV) and electrochemical impedance spectra (EIS) were conducted on the electrochemical workstation (CHI 760E). The EIS spectra were recorded with an amplitude of 5 mV in the frequency range from 0.01 Hz to 100000 Hz.

## 3 Results and discussions

**SCHEME 1:**
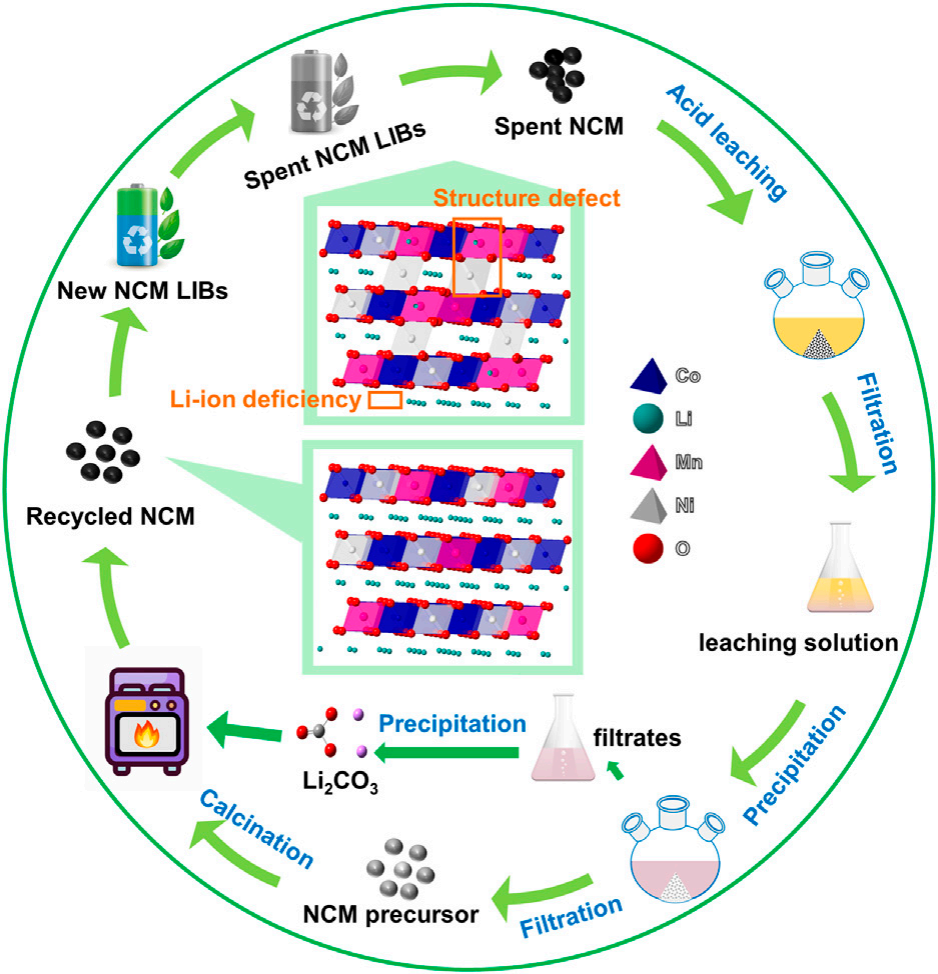
The schematic illustration of the regeneration process for spent NCM ternary cathode materials.

The recycling process for spent NCM ternary batteries is schematically demonstrated in Scheme 1. A lifecycle of the NCM battery starts from a newly produced battery, which come into retirement after long-time charge and discharge cycles. The spent battery was subjected to the pretreatment to get the spent NCM cathode powders. After the acid leaching, the metallic solution containing Li, Ni, Co, Mn ions were possessed.

The regeneration process starts from a precipitation reaction as shown in Eq. (1), with the oxalic acid solution as the precipitant to acquire the ternary metallic precursor:

$${\left[M\left(C_{6}H_{5}O_{7}\right)\right]}^{–}+H_{2}C_{2}O_{4}+2H_{2}O={\text{MC}}_{2}O_{4}∙2H_{2}O↓+C_{6}H_{5}O_{7}^{3–}+2H^{+}$$ (1)

After acid leaching, the resultant immersion solution was subjected to a co-precipitation reaction for 18 h by adding 0.55 mol/L oxalic acid solution. The ternary metal ions were transformed from the immersion solution into metal−oxalate salts (MC_2_O_4_∙2H_2_O, M = Ni, Co and Mn) (Refly et al., 2020). After filtration, we can obtain the precipitated product PNCM-18h, which was further used as the precursor of the calcination process. In addition, the filtrate can be used to get lithium carbonate (Li_2_CO_3_) by adding the Na_2_CO_3_ solution as the precipitant. The SEM images of PNCM-18 h in Supplementary Figure S1A and Supplementary Figure S1B reproduce the typical morphology of α-oxalate crystal, which was also reported in the related works (Cho et al., 2006; Park et al., 2010; Zhang et al., 2018). Supplementary Figure S1C displays the digital photo of the precipitate with a color of light green, due to the relatively high content of Ni in the NCM523 material. XRD was conducted on the precipitated product PNCM-18h, as shown in Supplementary Figure S2, in which the characteristic diffraction peaks can be indexed to the standard PDF card of oxalate dihydrate (PDF#25-0582). The inset of Supplementary Figure S2 provides the elemental analysis through the selected-area EDS scanning, equipped with the SEM. It indicates that Ni, Co and Mn elements co-exists in the precipitated oxalate with the atomic ratio of 9.28%, 3.48% and 6.04%, close to the original ratio of 5:2:3 in NCM523 material. This result proves that the oxalic acid solution can be used as the precipitant of Ni, Co and Mn.

For the regeneration of NCM, lithium carbonate (Li_2_CO_3_) was added into the mixture of metal−oxalate salts, followed with a solid-sate high-temperature calcination process. Before the calcination process, the element contents of Ni, Co, Mn, Li were measured by ICP, in order to make sure the dosage of the necessary element additive, thus restoring the elementary composition of the spent NCM to the pristine NCM. During the calcination process, the ternary precursor PNCM-18 h reacts with Li_2_CO_3_ to form LiMO_2_, the calcination process of which is as shown in Eq. (2).

$$4{\text{MC}}_{2}O_{4}∙2H_{2}O+2{\text{Li}}_{2}{\text{CO}}_{3}+3O_{2}=4{\text{LiMO}}_{2}+10{\text{CO}}_{2}↑+8H_{2}O↑$$ (2)

**FIGURE 1:**
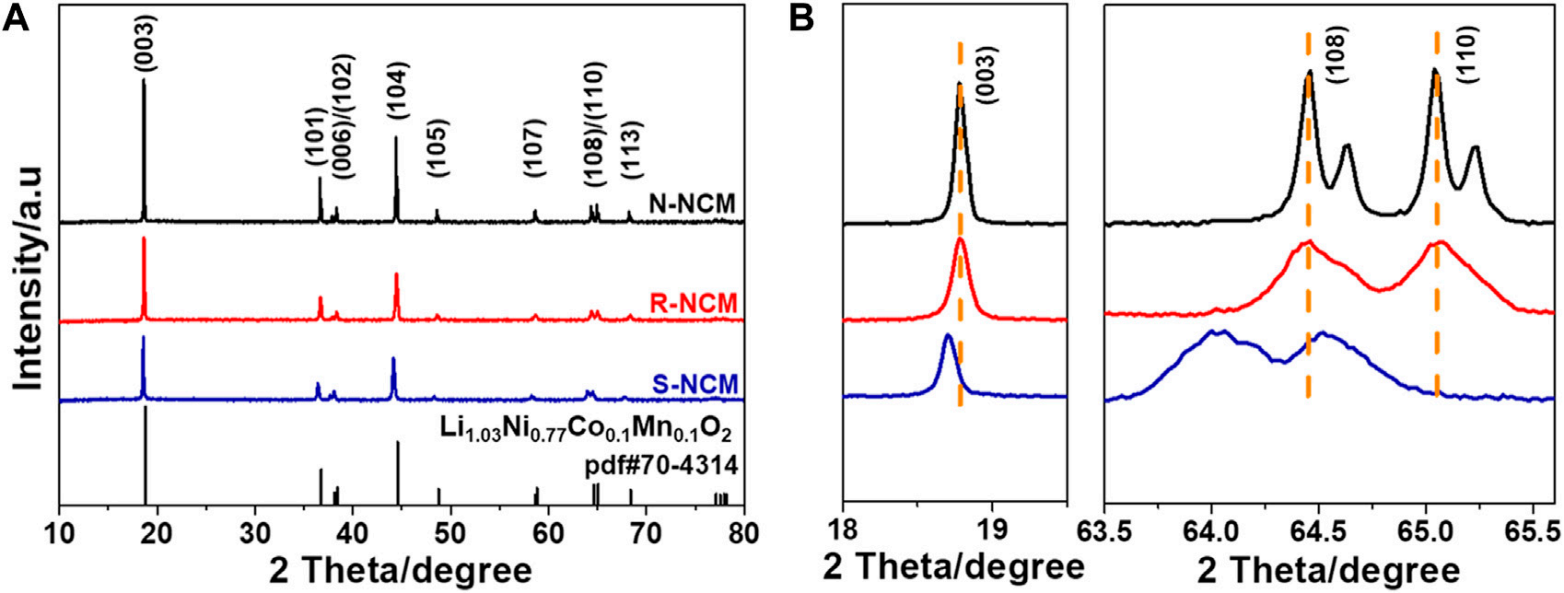
**(A)** XRD patterns of N-NCM, R-NCM and S-NCM; **(B)** magnified patterns in the ranges of 18.0°–19.5° and 64.0°–65.7°.

In order to analyze the crystalline structure, XRD was performed for commercial ternary new material N-NCM, regenerated ternary material R-NCM, and the pretreated waste ternary material S-NCM, with their patterns in Figure 1. The characteristic diffraction peaks of R-NCM and N-NCM in Figure 1A are consistent with the standard PDF card (PDF#70-4314), indicating a typical hexagonal α -NaFeO_2_-type layered structure. From Figure 1A, it is difficult to find the obvious differences between the S-NCM, R-NCM and N-NCM. However, after enlarging the spectra in the ranges of 18.0°–19.5°and 64.0°–65.7°, Figure 1B is obtained, showing that the (003) peak of S-NCM shifts to the left compared with that of N-NCM, while R-NCM is consistent with N-NCM. It is found that the (108) and (110) peaks of S-NCM have also shown the same shifting phenomenon while R-NCM is in accord with N-NCM. However, the twin peaks are only observed for N-NCM in Figure 1B, indicating that N-NCM has a highly ordered laminar structure (Deng et al., 2020). And the structure of R-NCM is still a little different from that of N-NCM. Rietveld refinement was conducted on the XRD patterns of the three samples to obtain the lattice parameters, as displayed in Table 1. It was reported that the c/a ratio is an indicator for layered structures (Deng et al., 2020). The c/a ratio of R-NCM is 4.964, which is higher than that of S-NCM and close to that of N-NCM, indicating that the regenerated material has regained a good layered structure. Moreover, the I_(003)_/l_(104)_ ratio is also an indicator for evaluating the Li^+^/Ni^2+^ disorder. The I_(003)_/L_(104)_ ratio of R-NCM is 1.657, which is much higher than that of the S-NCM (1.377), indicating that the cation mixing degree of the R-NCM is greatly reduced compared with S-NCM (Wu et al., 2015). It is considered that the lower the value of I_(006+ 102)_/I_(101)_, the more ordered the crystal structure of the material is. For a layered structure, the value is generally less than 0.5 (Mohanty et al., 2013). The I_(006+ 102)_/I_(101)_ value of the R-NCM is 0.49, which is much smaller than that of S-NCM (0.86) and slightly larger than that of N-NCM (0.42), indicating that R-NCM has a more ordered crystal structure than S-NCM. The XRD spectra and the derived lattice parameters show that the regenerated sample has recovered its crystal structure with good lamellar structure and high crystallinity.

**TABLE 1:** Lattice parameters of N-NCM, R-NCM and S-NCM through Rietveld refinement.

Sample	a (Å)	c(Å)	c/a	I_(003)_/I_(104)_	I_(006+102)_/I_(101)_
N-NCM	2.8654	14.2302	4.966	1.686	0.42
R-NCM	2.8674	14.2324	4.964	1.657	0.49
S-NCM	2.8627	14.1999	4.960	1.377	0.86

**FIGURE 2:**
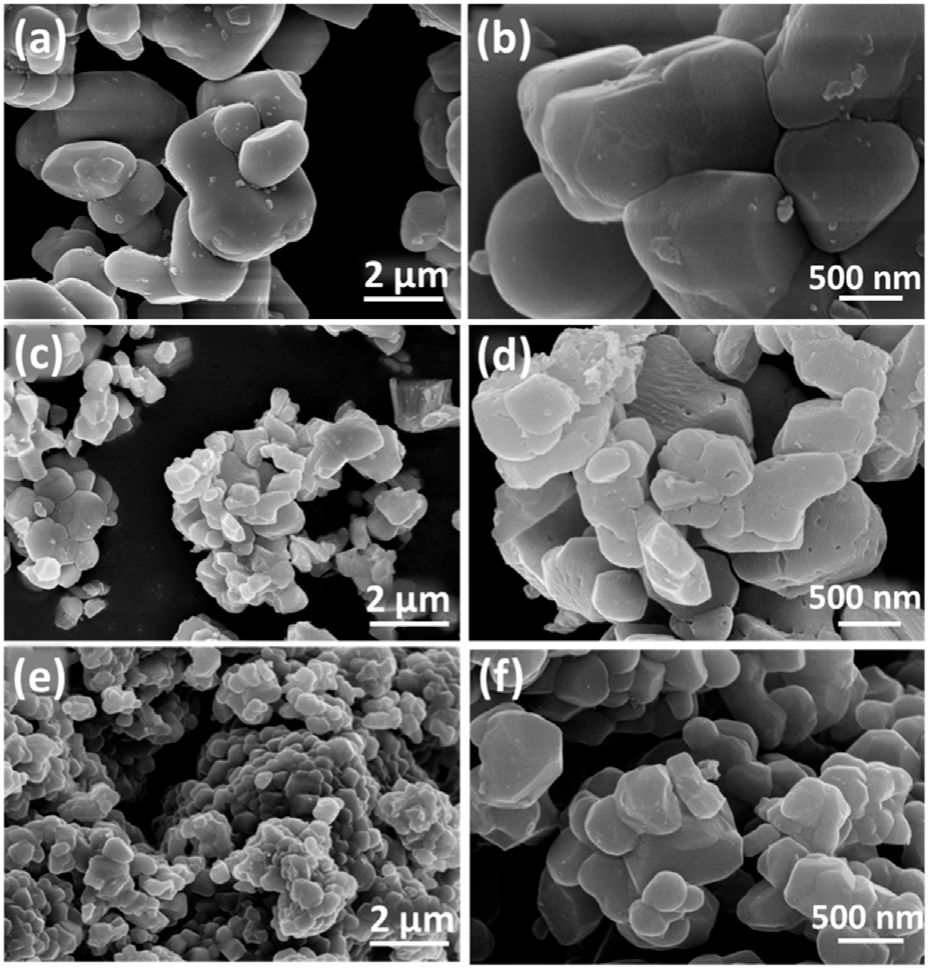
The SEM images of **(A, B)** N-NCM, **(C, D)** S-NCM, and **(E, F)** R-NCM.

In order to visualize the morphology and elemental distribution before and after regeneration, scanning electron microscopy (SEM) and EDS spectroscopy (EDS) tests were carried out on N-NCM, R-NCM and S-NCM, as shown in Figure 2. Figures 2A,B show the SEM images of N-NCM with smooth surface. The SEM images of S-NCM were shown in Figures 2C,D showing that the particle shape in the S-NCM becomes irregular and there are obvious cracks on the surface of the particles. This kind of surface structure change is probably ascribed to reiterative intercalation and de-intercalation of Li ions during the long-term discharge and charge process. However, in the R-NCM (Figures 2E,F), the surface becomes smooth and basically free of cracks. Therefore, the structure and the surface morphology of R-NCM was reconstructed through the regeneration process.

In order to further investigate the crystal structure of the regenerated material, high-resolution transmission electron microscopy (HRTEM) was carried out on the sample R-NCM, and the results are shown in Supplementary Figure S3. From Supplementary Figure S3A, the particle size of the regenerated material is around 0.5–1 μm. In Supplementary Figure S3B, it can be seen that the particles of the regenerated material displayed orderly lattice stripes, which indicates that the regenerated sample has a high degree of crystallinity, which is in line with the results of the XRD. In addition, the interplanar crystal spacing of R-NCM is calculated to be 0.48 nm, which is corresponded to the strongest diffraction peak of (003) crystal plane in the relative ternary NCM material (Zhang et al., 2019). EDS surface scanning was used to analyze the energy spectrum and elemental content of N-NCM, R-NCM and S-NCM, with the results shown in Supplementary Figure S4. The EDS analysis results show that all of three samples contain the major elements of O, Ni, Co, Mn and other elements such as C, F and Cu. According to the atomic percentages in the inset tables, the Ni: Co: Mn ratios of the materials are calculated as 0.46: 0.21: 0.32 for N-NCM, 0.46: 0.21: 0.33 for R-NCM and 0.44: 0.22: 0.34 for S-NCM, indicating that the elemental ratio of R-NCM is close to that of N-NCM. As can be seen from the EDS mapping scan in Supplementary Figure S5, the Ni, Co and Mn elements in R-NCM are uniformly distributed. In addition, there are no obvious changes in the elemental distribution of N-NCM, R-NCM and S-NCM. These results indicate that the contents of Ni, Co, Mn are steady but we need to care more about the content of Li. In order to measure the elemental contents of the three materials more accurately, the Li, Ni, Co and Mn contents in the materials were measured using an ICP-OES instrument, and the elemental molar ratios were further obtained. The results are shown in Table 2, which can be utilized as the basis of element complement. From Table 2, we can find that the contents of Ni, Co and Mn are stable, with no need of complement. However, S-NCM lost about 22% of Li, which may be caused by the irreversible reaction during long-term charging and discharging (Fan et al., 2020). Obviously, the regenerated sample R-NCM has regained the missing lithium, whose lithium content is higher than that in N-NCM. In addition, the content of Ni, Co, Mn in R-NCM can be accurately measured to be 0.497: 0.205: 0.298, which is very close to the ratio of 5: 2: 3.

**TABLE 2:** ICP-OES test results of N-NCM, R-NCM and S-NCM.

Sample	Element content (wt%)				Molar ratio
	Li	Ni	Co	Mn	
N-NCM	**7.56**	**29.27**	**11.83**	**16.51**	**Li**_**1.089**_ **Ni**_**0.499**_ **Co**_**0.201**_ **Mn**_**0.301**_
R-NCM	**7.90**	**29.15**	**12.11**	**16.37**	**Li**_**1.138**_ **Ni**_**0.497**_ **Co**_**0.205**_ **Mn**_**0.298**_
S-NCM	**5.39**	**28.76**	**12.11**	**16.73**	**Li**_**0.777**_ **Ni**_**0.490**_ **Co**_**0.205**_ **Mn**_**0.304**_

bold values mean the element weight percentage in the tested samples.

**FIGURE 3:**
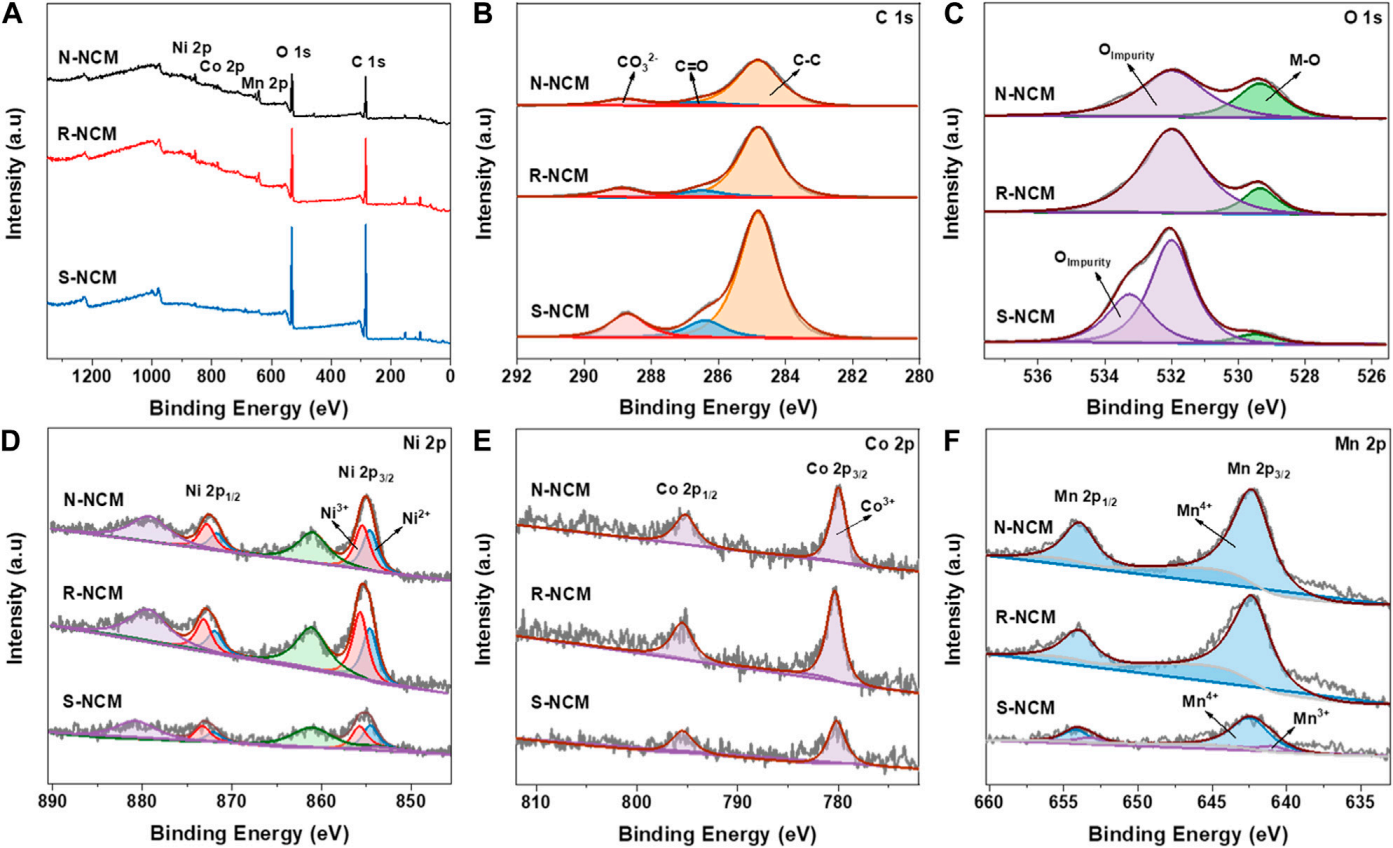
XPS spectra of N-NCM, R-NCM and S-NCM. **(A)** Survey scan; **(B)** C 1s spectra; **(C)** O 1s spectra; **(D)** Ni 2p spectra; **(E)** Co 2p spectra; **(F)** Mn 2p spectra.

X-ray photoelectron spectroscopy (XPS) was used to study the chemical valence states of C, O, Ni, Co and Mn on the surfaces of N-NCM, R-NCM and S-NCM, with the results shown in Figure 3. The survey spectrum of in Figure 3A shows that the three samples consist of Ni, Co Mn, C, and O. The C 1s spectrum of Figure 3B can be divided into three corresponding peaks CO_3_^2-^ (288.8 eV), C=O (286.5 eV) and C-C (284.8 eV) (Huang et al., 2019). The peak areas of the three peaks for CO_3_^2-^ (288.8 eV) are calculated to be 11.6%, 10.9% and 13.3%, respectively. The O 1s spectrum of Figure 3C can be fitted into two peaks, including impurity oxygen (531.9 eV, 533.3 eV) and lattice oxygen (529.5 eV), respectively (Liu et al., 2007; Chen et al., 2015), where the impurity oxygen is mainly ascribed to the impurity layer, from the adsorbed hydroxyl and carbonate species. The lattice oxygen is the O^2-^ in M-O (288.8 eV) (M = Ni, Co, Mn) bonds of NCM material (Liu et al., 2013). It is clear that the percentage of impurity oxygen in R-NCM is lower than that of the S-NCM, but still higher than of that of N-NCM, indicating the existing difference between R-NCM and N-NC. The XPS spectra of Ni 2p are shown in Figure 3D. The binding energy peaks of Ni 2p3/2 and 2p1/2 are around 854.2 and 873.3 eV, respectively, and the satellite peaks are located at 861 eV and 879.1 eV, respectively (Li et al., 2014). The Ni 2p_3/2_ peaks can be split into two fitted peaks, i.e., Ni^2+^ (854.6 eV) and Ni^3+^ (855.7 eV). We can find by peak area calculation that the Ni^2+^/Ni^3+^ value of R-NCM is lower than that of S-NCM, but similar to that of N-NCM, indicating the lower content of Ni^3+^ in S-NCM. It is known that the Ni^3+^ is favorable to reduce the cation mixing and improve the electrochemical performance (Zhang et al., 2018). The XPS spectra of Co 2p are shown in Figure 3E, in which the binding energy peaks at around 780.0 eV and 795.1 eV are corresponded to Co 2p_3/2_ and 2p_1/2_ species for Co^3+^ (Hao et al., 2019). It is found that the Co 2p XPS spectra of the three samples were basically not different. Figure 3F shows the XPS spectra of Mn 2p. The fitted peaks at 642.3 eV and 654.2 eV correspond to Mn^4+^, and the fitted peaks at 640.9 eV and 652.8 eV correspond to Mn^3+^ (Deng et al., 2020). The presence of Mn^3+^ in S-NCM is due to the transition from a layered structure to a spinel structure, probably to compensate the change of charge. In addition, the signal of Mn^3+^ decrease in R-NCM, which is similar structure to N-NCM.

**FIGURE 4:**
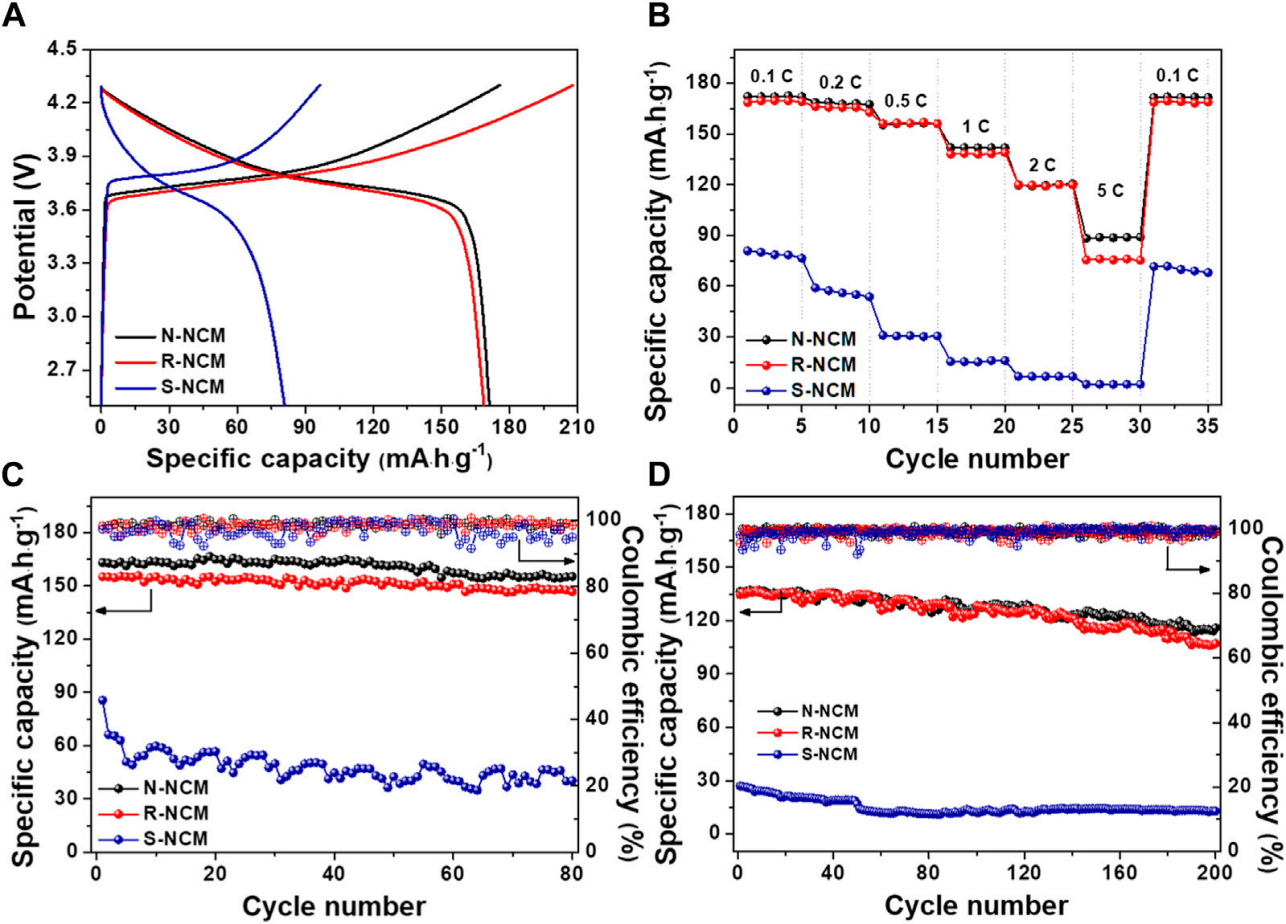
The battery performances for N-NCM, R-NCM and S-NCM. **(A)** First charge/discharge curves at 0.1 C; **(B)** discharge performance at different rates; **(C)** cycle performance and Coulombic efficiency at 0.2 C for 80 cycles; **(D)** cycle performance and Coulombic efficiency at 1 C for 200 cycles.

In order to testify the effectivity of the regeneration process for the spent NCM materials, N-NCM, R-NCM and S-NCM were used as the cathode materials to assemble the 2032-type coin cells for the electrochemical performance evaluation. As shown in Figure 4, the batteries based on the three materials were charged and discharged at the voltage range of 2.5–4.3 V. Figure 4A shows the first-round charge/discharge curve of the battery at the rate of 0.1 C. The discharge curve of S-NCM is obviously steeper and the voltage plateau is relatively low. In addition, the charge/discharge specific capacity is lower, which is partly due to the loss of lithium, the change of crystalline structure and the surface transition during the long-term cycling process. The charge/discharge curves of R-NCM are longer and smoother than those of S-NCM, but very close to those of N-NCM. The discharge specific capacity of the R-NCM is 168.6 mAh ∙ g^-1^, very close to 172.1 mAh ∙ g^-1^ of N-NCM and much higher than 80.9 mAh ∙ g^-1^ for S-NCM. This result indicates that the capacity has been effectively recovered after the regeneration process. Figure 4B exhibits the discharge curves of the battery at 0.1–5 C. The specific discharge capacity of R-NCM is much higher than that of S-NCM at each rate, and close to N-NCM. Figure 4C shows the cycling stability of the cells at a current density of 0.2 C. The initial discharge specific capacity of R-NCM is 155.1 mAh ∙ g^-1^, which is a little lower than 163.2 mAh ∙ g^-1^ for N-NCM. After 80 cycles, the capacity retentions of N-NCM and R-NCM reach 95% and 94%, respectively, while that of S-NCM is only 47%. The Coulombic efficiency corresponding to the *y*-axis on the right-hand side shows that the charge and discharge efficiencies of N-NCM and R-NCM are always stable for more than 95%, whereas the Coulombic efficiency of S-NCM is unstable. The batteries were subjected to long-cycle tests at a higher current density, as shown in Figure 4D. At 1 C current density, R-NCM demonstrates comparable discharge specific capacity of 134.6 mAh ∙ g^-1^ to 136.5 mAh ∙ g^-1^ for N-NCM. After 200 cycles, the capacity retention rate of R-NCM is 80%, close to 85% for N-NCM, and the Coulombic efficiency is above 98%. At the high current, the initial specific discharge capacity of S-NCM is only 20% that of R-NCM. After a long cycle, the specific discharge capacity of S-NCM decreases to only 12.8 mAh ∙ g^-1^. These tests show that the R-NCM has excellent electrochemical properties. The regenerated samples obtained at various heat-treatment temperatures were also utilized to assemble the coin cell. As shown in Supplementary Figure S6, R-NCM-850 shows the largest charge/discharge capacity. Moreover, R-NCM-850 also displays the superior rate capability and cyclic stability to other samples.

To further investigate the kinetic properties and redox processes of the samples before and after regeneration, cyclic voltammetry (CV) tests were performed on N-NCM, R-NCM and S-NCM. Supplementary Figures S7A–C show the CV curves for the three materials in the voltage range of 2.5–4.3 V at a sweep rate of 0.1 mV ∙ s^-1^ for the first three CV cycles. The CV curves of all three materials show one oxidation peak and one reduction peak, which correspond to the redox reactions of Ni^2+^/Ni^4+^ (Chen et al., 2019). The potential difference (ΔE) of the redox peaks for the three materials in the first cycle were 0.382, 0.401, and 0.411 V, respectively. These values reflect the polarization degree of the battery. The larger value of ΔE means the greater degree of polarization, which is related to the higher degree of cation mixing (Li et al., 2014). Therefore, we find that S-NCM has the most serious polarization, and R-NCM is similar to N-NCM. It is also found that after the first cycle, the CV curves of the three samples are gradually stabilized, and ΔE decreases. The CV curves in the third cycle for the three materials are compared in Supplementary Figure S7D, and the ΔE value of R-NCM is 0.252 V, close to that of the N-NCM (0.246 V), and lower than that of S-NCM (0.294 V). The larger ΔE value of S-NCM than N-NCM and R-NCM indicates that the charge/discharge specific capacity of N-NCM and R-NCM may be higher than that of S-NCM (Deng et al., 2020), which is consistent with the battery performance analysis in Figure 4A. All the electrochemical tests prove that the regenerated NCM material exhibits a similar battery performance compared to that of commercial new NCM material. The EIS spectra were recorded to study the charge transfer capacity at the interface for S-NCM, N-NCM, and R-NCM. The fitted curves were displayed in Supplementary Figure S8, with the equivalent circuit diagram illustrated at the inset. The semicircle at the intermediate frequency region indicates the charge transfer resistance (Rct), corresponding to electrochemical reaction at the electrode interface. It is obvious that N-NCM (72.8 Ω) and R-NCM (106.1 Ω) exhibits the similar Rct values, much lower than that of S-NCM (277.0 Ω). The higher Rct value of S-NCM is probably due to change of surface structure and the deficiency of Li.

## 4 Conclusion

In a summary, an effective and facile solid-state-calcination strategy was developed to regenerate the waste NCM cathode materials in spent LIBs. The regenerated material was characterized by physicochemical characterizations, confirming that the hexagonal α-NaFeO_2_-type layered structure was recovered and the elementary constitute resorted to the pristine state. Due to recovery in material properties, the regenerated material can possess excellent electrochemical performance, very close to that of commercial NCM. This kind of method is easy to enlarge production for the industrial recycling of spent NCM LIBs.

## Data Availability

The raw data supporting the conclusions of this article will be made available by the authors, without undue reservation.
