# Supplementary material for: Research on the facile regeneration of degraded cathode materials from spent LiNi0.5Co0.2Mn0.3O2 lithium-ion batteries
Source: Front Chem. 2024 Apr 30;12:1400758. doi: 10.3389/fchem.2024.1400758 (PMC11091315; doi:10.3389/fchem.2024.1400758)
Supplement: Supplementary file 1 [file DataSheet1.docx]

**Research on the facile regeneration of degraded cathode materials from spent LiNi_0.5_Co_0.2_Mn_0.3_O_2_ lithium-ion batteries**

Chen Yang, Yujia Hao, Jiayi Wang, Mingdao Zhang, Li Song*, Jiaan Qu*

*School of Environmental Science and Engineering, Jiangsu Key Laboratory of Atmospheric Environment Monitoring and Pollution Control, Jiangsu Collaborative Innovation Center of Atmospheric Environment, Nanjing University of Information Science & Technology, Nanjing, 210044, Jiangsu, P. R. China.*

E-mail: songli@nuist.edu.cn; 000968@nuist.edu.cn


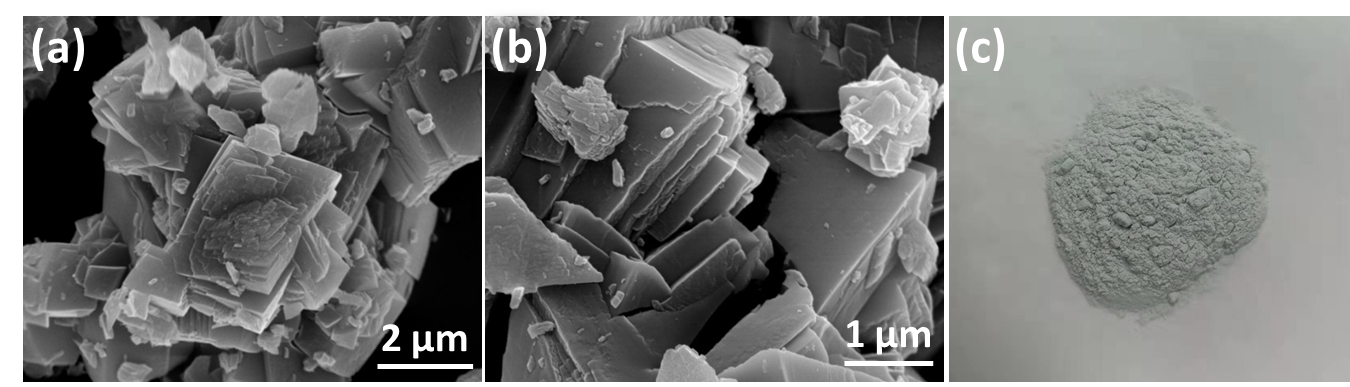


**Figure S1.** (a, b) SEM images and (c) the digital picture of the precipitated product PNCM-18h


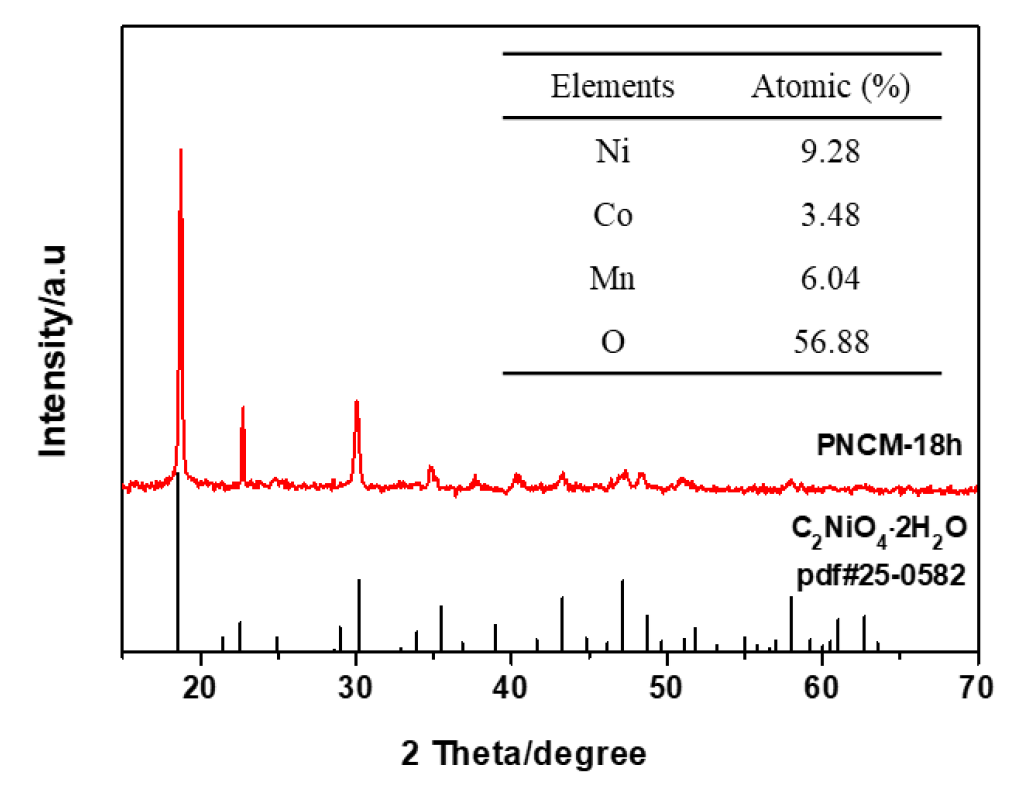


**Figure S2.** The XRD pattern of the precipitated product PNCM-18h. The inset is the EDS analysis accompanied with SEM.


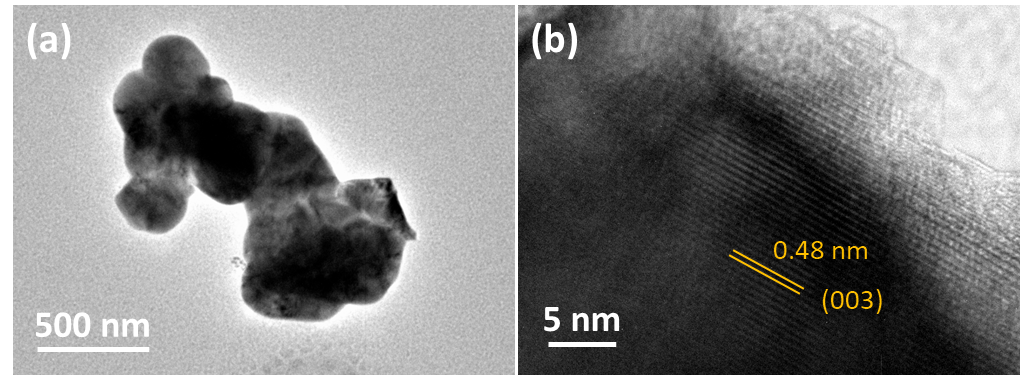


**Figure S3.** Transmission electron microscopy of recycled material R-NCM


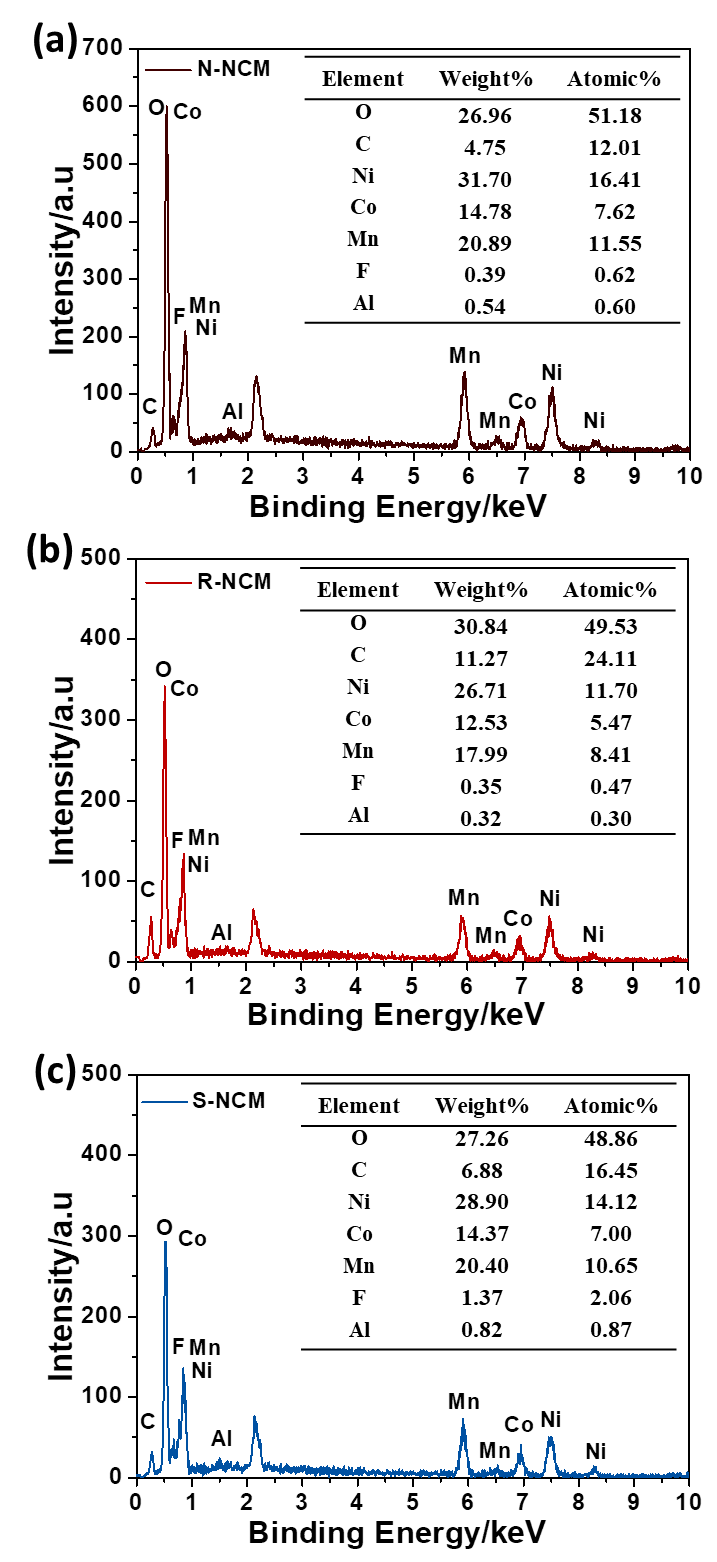


**Figure S4.** The EDS analysis of N-NCM, R-NCM and S-NCM, equipped on the SEM.


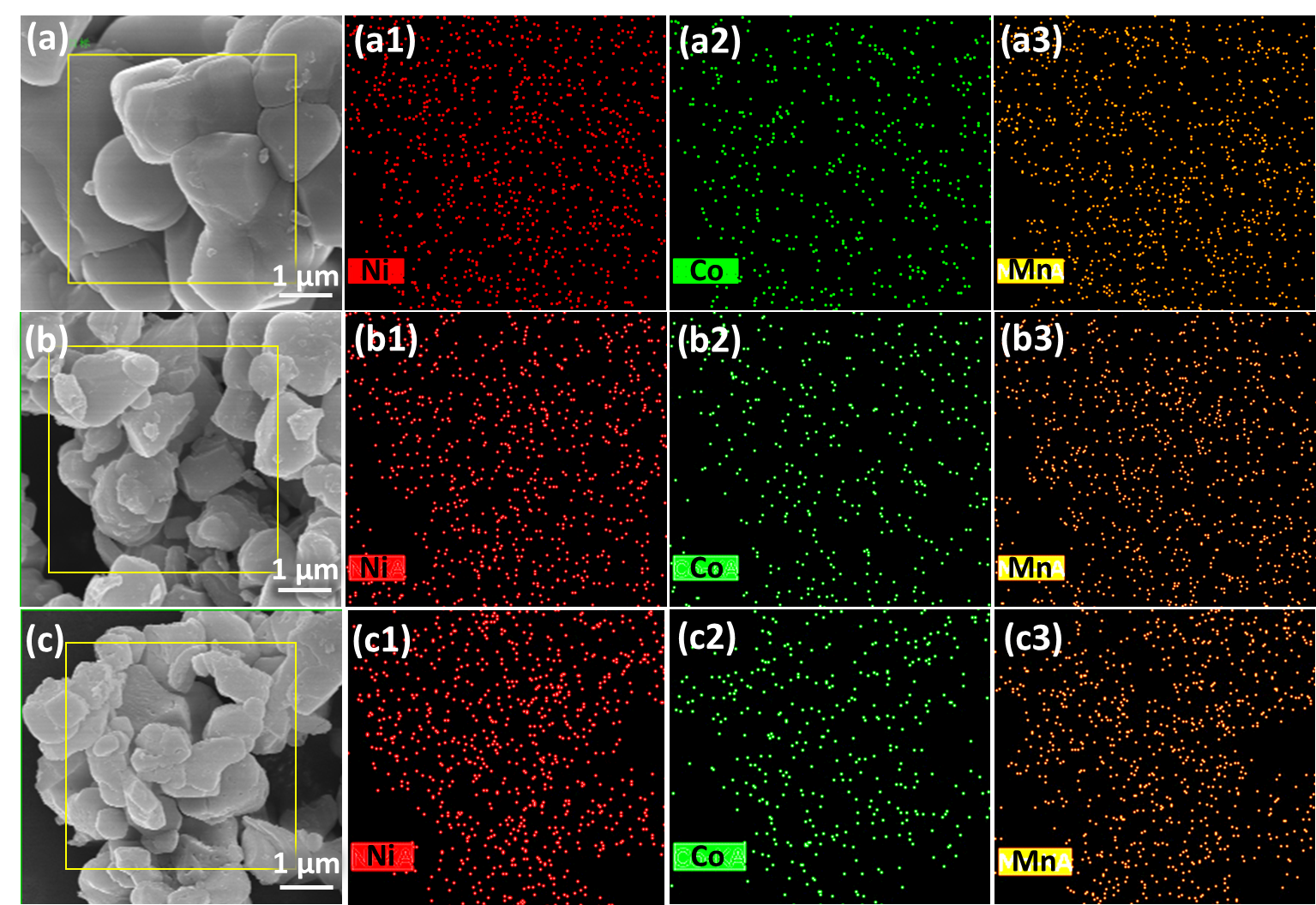


**Figure S5.** The EDS mapping scan analysis of N-NCM, R-NCM and S-NCM, equipped on the SEM.


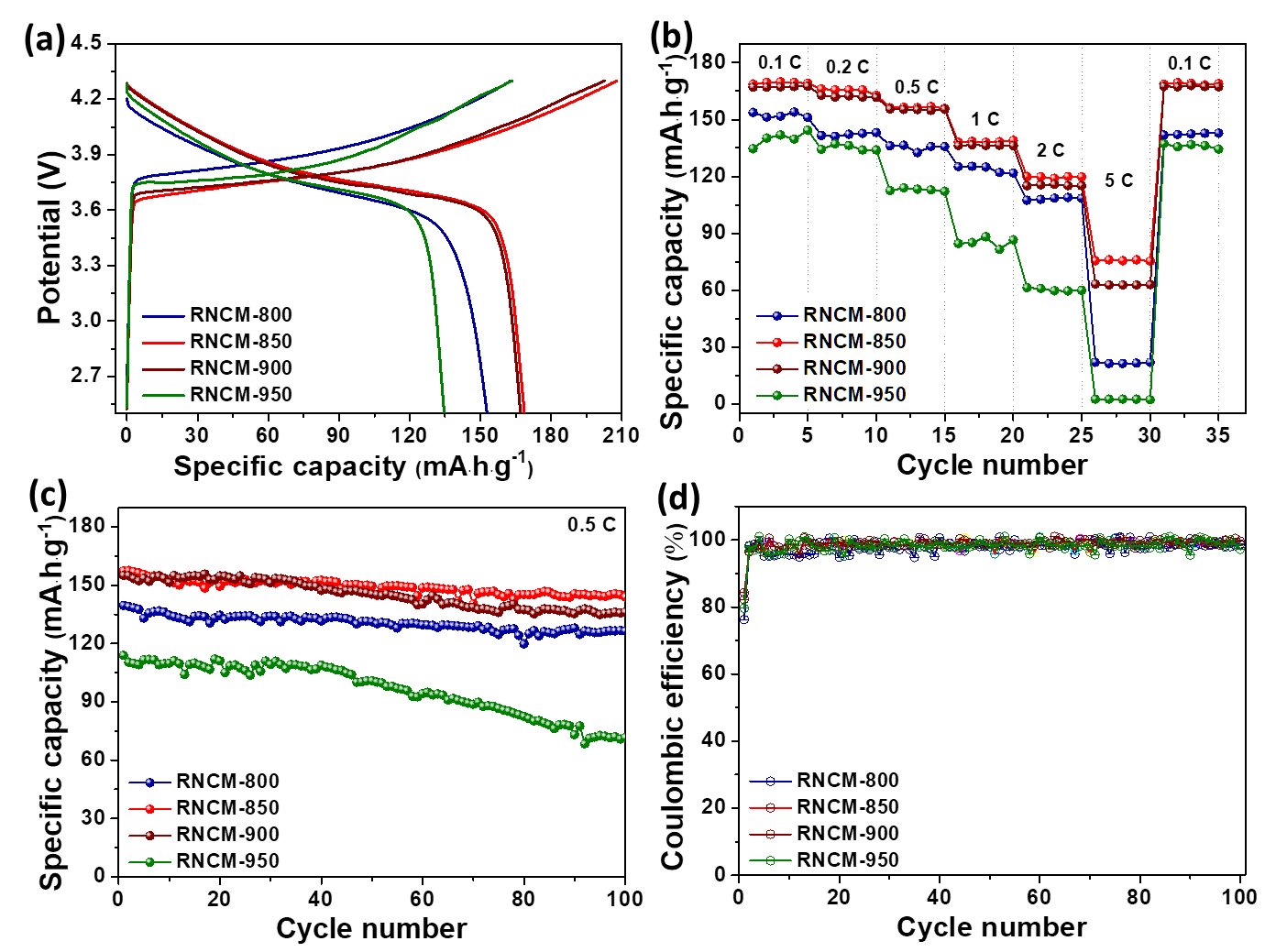


**Figure S6.** The battery performances for R-NCM at various calcination temperatures.

(a) first charge/discharge curves at 0.1 C; (b) discharge performance at different rates; (c) cycle performance and coulombic efficiency at 0.5 C; (d) cycle performance and coulombic efficiency at 1 C.


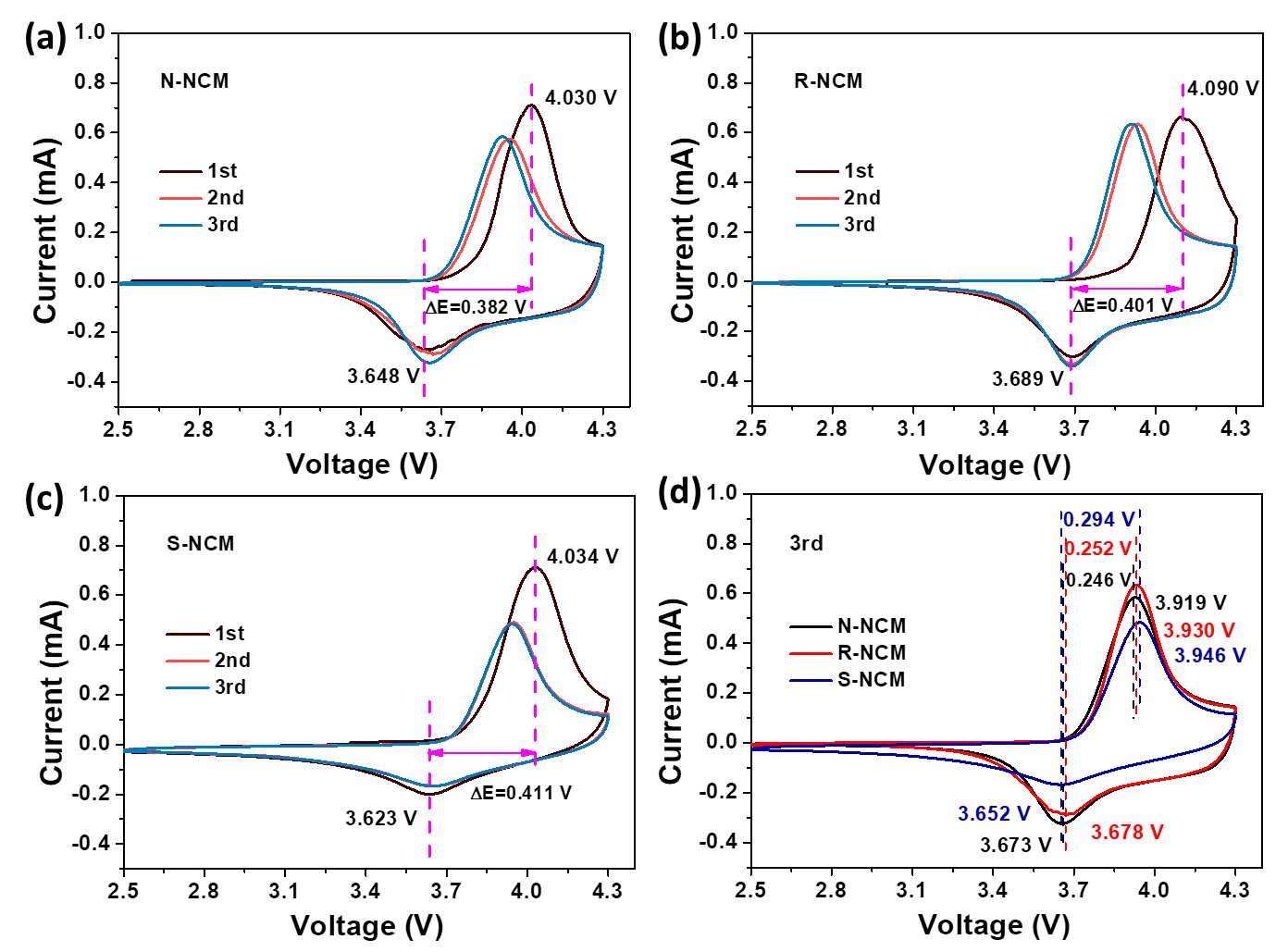


**Figure S7.** CV curves of (a) N-NCM, (b) R-NCM and (c) S-NCM in the voltage range of 2.5-4.3 V; (d) CV curves of the third turn of the three materials in the voltage range of 2.5-4.3 V.


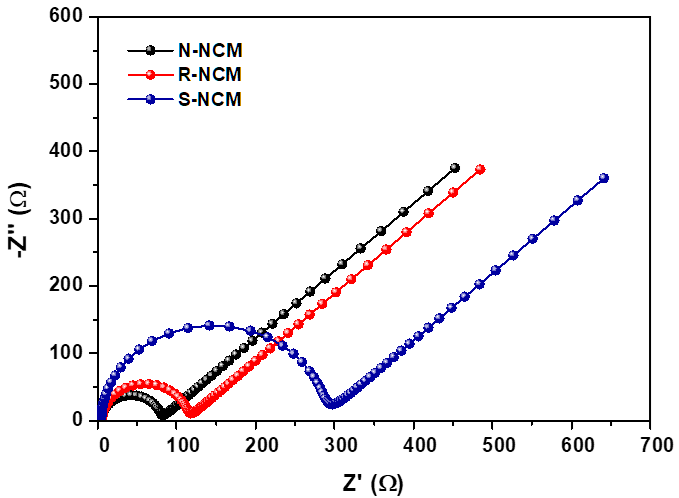


**Figure S8.** The EIS spectra for N-NCM, (b) R-NCM and (c) S-NCM
